# Supplementary material for: Measurement Properties of the Dutch Multifactor Fatigue Scale in Early and Late Rehabilitation of Acquired Brain Injury in Denmark
Source: J Clin Med. 2023 Mar 29;12(7):2587. doi: 10.3390/jcm12072587 (PMC10094862; doi:10.3390/jcm12072587)
Supplement: Supplementary file 1 [file jcm-12-02587-s001.zip › jcm-2233776-supplementary.pdf]

ONLINE SUPPLEMENTARY MATERIAL

Measurement Properties of the Dutch Multifactor Fatigue Scale in Early and Late Rehabilitation of Acquired Brain Injury in Denmark

Correlation matrices 1

Item descriptive statistics 4

Standardized factor loadings 9

Post hoc measurement invariance analyzes 12

Correlation matrices

Summary data (inter-item correlation matrices) of the full sample (N = 149) for subscales of the Dutch Multifactor Fatigue Scale (DMFS) are provided in Table S1–S5. Correlations were calculated using the polychoric correlation coefficient (using the *psych* package [47]), as appropriate for ordinal data.

Table S1. Impact of Fatigue: Inter-Item Correlations (Polychoric)

Item	1	6	13	20	22	24	26	29	33	35	38
1	1										
6	.58	1									
13	.59	.54	1								
20	.59	.46	.45	1							
22	.52	.55	.51	.47	1						
24	.14	.23	.17	.32	.23	1					
26	.58	.62	.55	.53	.60	.34	1				
29	.47	.59	.33	.47	.49	.10	.50	1			
33	.66	.66	.49	.55	.58	.31	.61	.52	1		
35	.39	.51	.44	.40	.49	.39	.52	.31	.63	1	
38	.53	.63	.46	.50	.57	.15	.66	.60	.74	.46	1

Table S2. Signs and Direct Consequences of Fatigue: Inter-Item Correlations (Polychoric)

Item	4	7	11	15	19	21	23	28	31
4	1								
7	.37	1							
11	.35	.27	1						
15	.46	.24	.40	1					
19	.13	.29	.25	.17	1				
21	.35	.47	.29	.35	.32	1			
23	.19	.31	.21	.27	.20	.34	1		
28	.27	.37	.14	.25	.30	.56	.32	1	
31	.40	.46	.48	.32	.40	.38	.30	.48	1

Table S3. Mental Fatigue: Inter-Item Correlations (Polychoric)

Item	3	10	17	27	32	34	37
3	1						
10	.25	1					
17	.31	.45	1				
27	.39	.41	.58	1			
32	.21	.39	.45	.65	1		
34	.34	.42	.52	.69	.51	1	
37	.46	.51	.59	.59	.42	.51	1

Table S4. Physical Fatigue: Inter-Item Correlations (Polychoric)

Item	5	9	14	18	25	30
5	1					
9	.70	1				
14	.31	.40	1			
18	.31	.31	.09	1		
25	.27	.27	.37	.18	1	
30	.40	.38	.50	.38	.40	1

Table S5. Coping with Fatigue: Inter-Item Correlations (Polychoric)

Item	2	8	12	16	36
2	1				
8	-.11	1			
12	.22	.05	1		
16	.02	.37	-.10	1	
36	.20	.40	.10	.36	1

Item descriptive statistics

Descriptive statistics for items on the subscales of DMFS in the full sample are provided in Table S6. Figure S1 shows the item response distribution on all items by rehabilitation setting (community-based vs. sub-acute). For each item, all response categories were observed in both groups. Figure S2 provides monotonicity plots in the full sample, i.e., relationships between each item and the corrected total score.

Table S6. Item Descriptive Statistics on Dutch Multifactor Fatigue Scale in Acquired Brain Injury

Item		Univariate statistics					Correlations	
no.	Content, abbreviated	*n*	*M*	*SD*	Floor, %	Ceiling, %	Inter-item, range r_{pc}	Item-total, r_{ps}
				Impact of Fatigue				
1	Often tired	149	4.17	1.08	4.7	49.7	.14 to .66	.74
6	Do what I want	149	3.73	1.39	11.4	40.3	.23 to .66	.77
13	Overcome	149	3.86	1.38	12.1	45.0	.17 to .59	.62
20	Tired every day	148	4.05	1.21	6.1	48.6	.32 to .59	.65
22[a]	Easily get over	149	3.09	1.38	14.1	21.5	.23 to .60	.68
24[a]	Don't need rest	148	4.00	1.35	10.8	52.0	.10 to .39	.27
26	Suffer	149	2.91	1.59	29.5	24.8	.34 to .66	.76
29	Serious problem	149	3.26	1.53	21.5	29.5	.10 to .60	.59
33	Affects life	149	3.26	1.53	22.8	28.2	.31 to .74	.81
35	Can't go further	149	3.61	1.42	12.1	37.6	.31 to .63	.60
38	Suffer terribly	149	2.68	1.47	30.2	16.1	.15 to .74	.73
Mean		-	3.51	1.39	-	-	.48	.66
				Signs and Direct Consequences of Fatigue				
4	In the afternoon	149	4.06	1.21	5.4	51.7	.13 to .46	.50
7	Emotional issues	149	3.09	1.43	19.5	22.8	.24 to .47	.54
11[a]	Recover easily	149	3.19	1.40	14.1	24.8	.14 to .48	.44
15	Others notice	149	3.44	1.35	12.1	26.2	.17 to .46	.46
19	Headache	149	2.40	1.53	45.0	15.4	.13 to .40	.40
21	React emotionally	149	3.57	1.38	12.8	33.6	.29 to .56	.58
23	Let thoughts go	149	3.19	1.42	18.1	23.5	.19 to .34	.40
28	Regret things said	149	2.53	1.48	38.9	12.8	.14 to .56	.51
31	Bother next day	149	3.40	1.48	16.8	32.2	.30 to .48	.62
Mean		-	3.21	1.41	-	-	.32	.49
				Mental Fatigue				
3[a]	Conversations	149	2.69	1.31	24.2	8.1	.21 to .46	.39
10	Thinking	149	3.40	1.39	14.1	26.2	.25 to .51	.51

Item		Univariate statistics					Correlations	
no.	Content, abbreviated	*n*	*M*	*SD*	Floor, %	Ceiling, %	Inter-item, range r_{pc}	Item-total, r_{ps}
17	Stimulation	149	4.05	1.25	8.1	51.0	.31 to .59	.66
27	Concentrating	148	4.03	1.19	4.7	47.3	.39 to .69	.78
32	Make mistakes	149	3.79	1.25	8.1	36.9	.21 to .65	.57
34	Complaints	149	3.94	1.31	9.4	46.3	.34 to .69	.67
37	Cannot think	149	3.20	1.50	19.5	28.2	.42 to .59	.67
Mean		-	3.59	1.31	-	-	.46	.61
				Physical Fatigue				
5[a]	Physically fit	149	2.79	1.36	22.8	13.4	.27 to .70	.56
9[a]	Good condition	149	2.63	1.38	28.2	12.1	.27 to .70	.58
14[a]	Wake up rested	149	2.71	1.46	26.2	18.1	.09 to .50	.45
18	Physical exertion	148	3.72	1.43	12.2	43.2	.09 to .38	.32
25	Body aches	149	2.13	1.44	51.7	11.4	.18 to .40	.40
30	Little energy	149	3.37	1.41	14.8	28.2	.38 to .50	.57
Mean		-	2.89	1.41	-	-	.35	.48
				Coping with Fatigue				
2[a]	Plan rest	149	2.79	1.43	24.2	17.4	-.11 to .22	.14
8	Get tired out	148	2.84	1.43	24.3	16.9	-.11 to .40	.30
12	Finish doings	149	3.56	1.29	9.4	28.2	-.10 to .22	.14
16[a]	Avoid overtired	149	2.91	1.33	16.8	14.8	-.10 to .37	.28
36	Get overtired	149	3.21	1.47	18.8	25.5	.10 to .40	.47
Mean		-	3.06	1.39	-	-	.15	.27

Note. Analyses were conducted in the full sample (N = 149). r_{pc} = polychoric correlation coefficient. r_{ps} = polyserial correlation coefficient.

[a] The item is reverse keyed, i.e. worded in positive terms, and was reverse coded prior to analyses.

Figure S1. Item Response Distribution on the Dutch Multifactor Fatigue Scale by Rehabilitation Setting

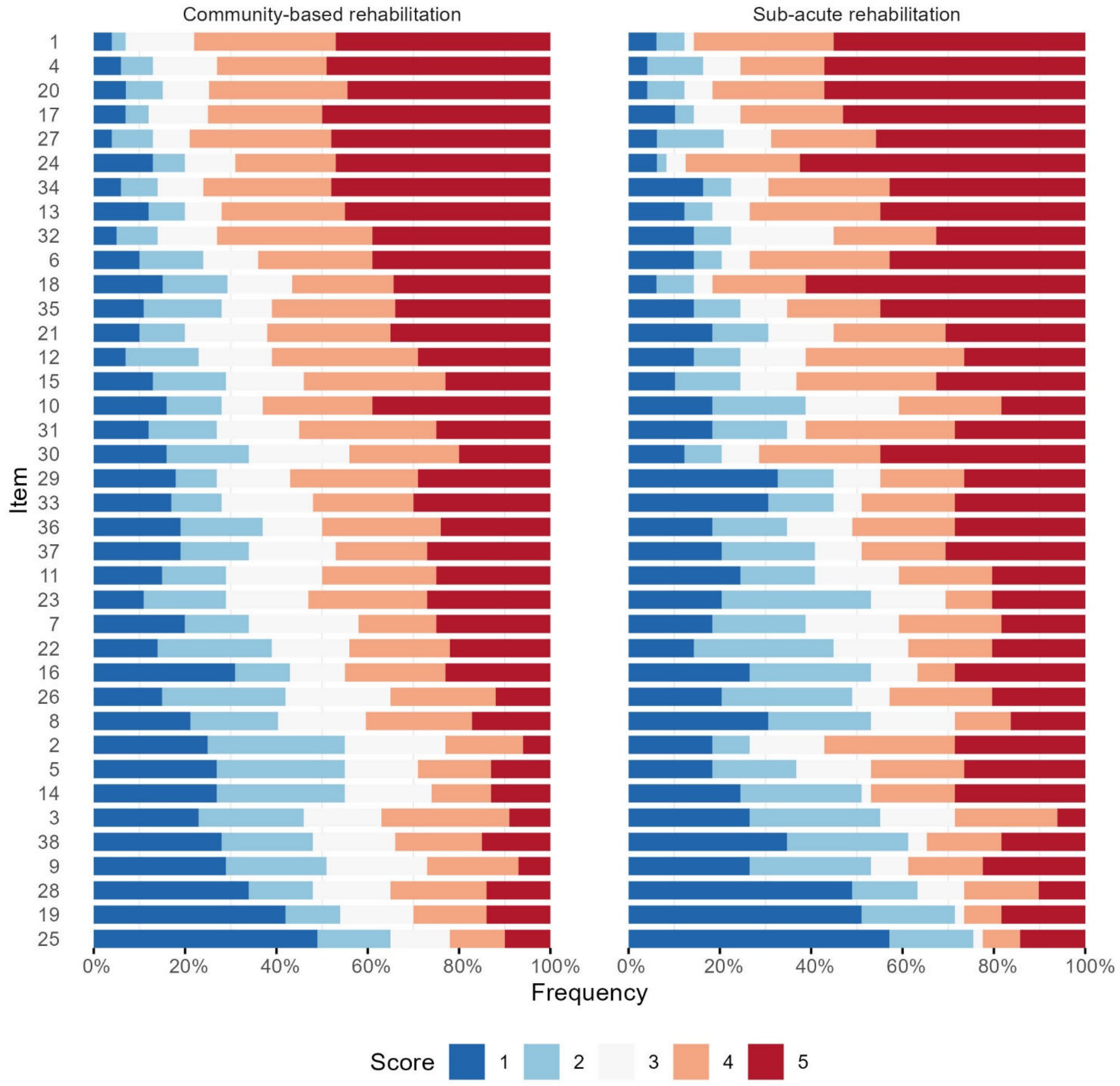

Note. Reverse keyed items, i.e. negatively worded items, were reverse coded prior to analysis.

Figure S2. Monotonicity Plots in the Full Sample

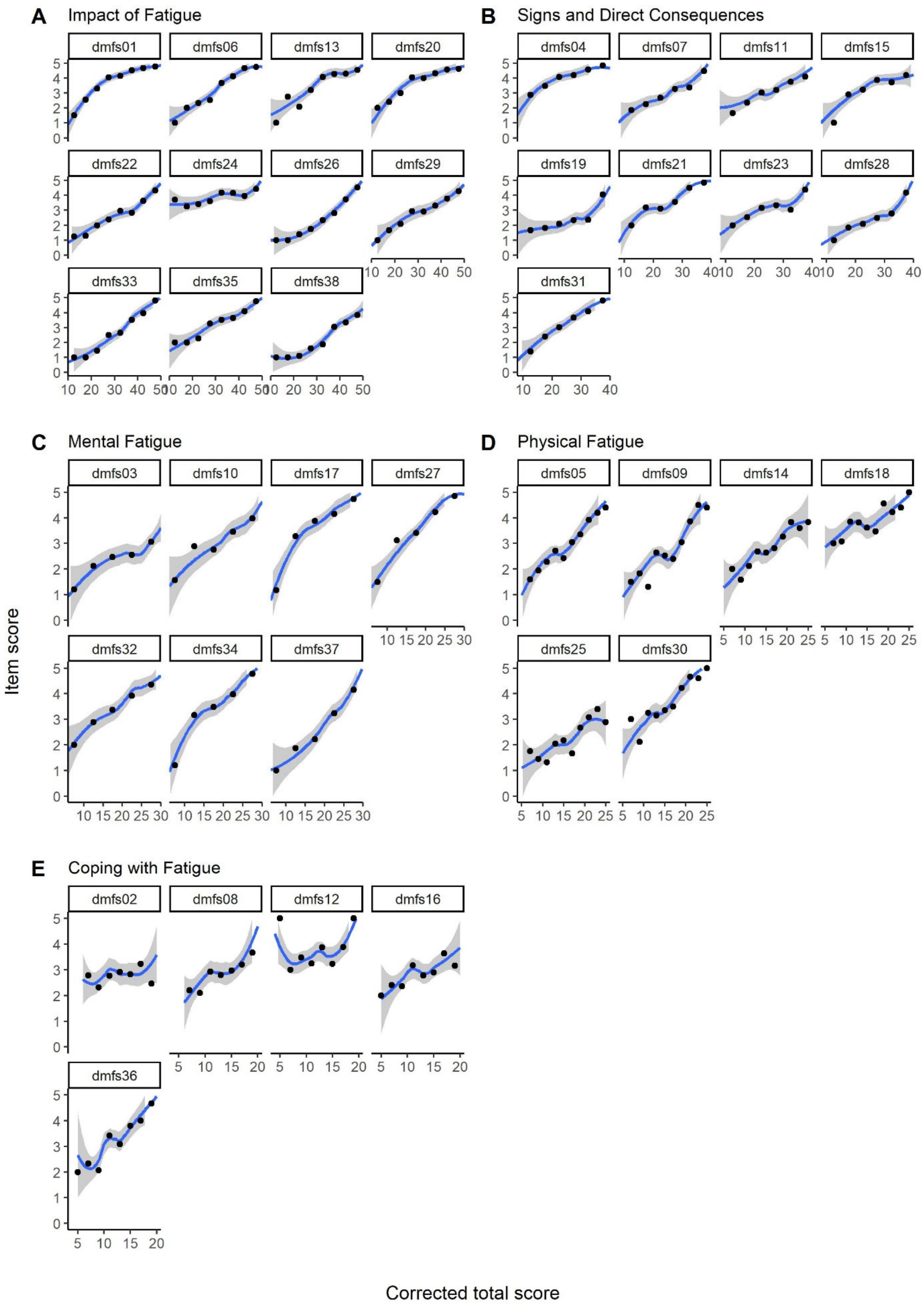

Note. The corrected total score (corrected for item overlap) was used as a proxy for the latent variable. Points represent binned means. Smooth curves were calculated using locally estimated scatterplot smoothing (loess). Grey areas display the 95% confidence interval.

Standardized factor loadings

Table S7 reports standardized factor loadings of measurement models in the full sample, and Table S8 reports standardized factor loadings of the multi-group factor models that were fitted in measurement invariance testing. Threshold estimates are not reported.

Table S7. Standardized Factor Loadings in Full Sample (*N* = 149)

Item	Est	*SE*	z	*p*
		Impact of Fatigue		
1	0.75	0.05	15.51	< .001
6	0.78	0.04	19.12	< .001
13	0.66	0.06	11.44	< .001
20	0.67	0.06	12.01	< .001
22	0.73	0.04	17.08	< .001
24	0.33	0.08	3.99	< .001
26	0.79	0.04	20.40	< .001
29	0.66	0.05	12.83	< .001
33	0.86	0.03	27.83	< .001
35	0.65	0.06	11.19	< .001
38	0.81	0.03	24.46	< .001
		Signs and Direct Consequences of Fatigue		
4	0.57	0.08	7.45	< .001
7	0.62	0.06	10.44	< .001
11	0.53	0.07	7.95	< .001
15	0.53	0.07	7.90	< .001
19	0.45	0.08	5.46	< .001
21	0.69	0.05	14.28	< .001
23	0.46	0.07	6.58	< .001
28	0.63	0.06	10.52	< .001
31	0.72	0.05	14.27	< .001
		Mental Fatigue		
3	0.47	0.07	6.68	< .001
10	0.57	0.06	9.75	< .001
17	0.71	0.05	12.91	< .001
27	0.86	0.03	28.89	< .001
32	0.68	0.05	13.76	< .001
34	0.76	0.04	17.75	< .001
37	0.73	0.05	15.00	< .001
		Physical Fatigue		
5	0.79	0.05	17.37	< .001
9	0.80	0.05	16.26	< .001
14	0.57	0.06	8.86	< .001
18	0.41	0.08	5.22	< .001
25	0.45	0.09	5.26	< .001
30	0.63	0.06	11.02	< .001
		Coping with Fatigue		
2	-0.08	0.10	-0.85	.395
8	-0.61	0.08	-7.60	< .001
12	-0.05	0.09	-0.61	.544
16	-0.56	0.08	-6.95	< .001
36	-0.67	0.09	-7.13	< .001

Table S8. Standardized Factor Loadings Across Rehabilitation Settings

Item	Community-based (n = 100)		Equality constrained		Sub-acute (n = 49)	
	Est	*SE*	Est	*SE*	Est	*SE*
			Impact of Fatigue–10 (w/o Item 24)			
1			$.77^{***}$	.05		
6			$.85^{***}$	.04		
13			$.66^{***}$	.07		
20			$.71^{***}$	.06		
22			$.74^{***}$	.05		
26			$.84^{***}$	.04		
29			$.70^{***}$	.05		
33			$.89^{***}$	.03		
35			$.68^{***}$	.06		
38			$.84^{***}$	.03		
			Signs and Direct Consequences of Fatigue			
4			$.57^{***}$	.08		
7	$.70^{***}$	.07			$.30^{**}$	.10
11			$.53^{***}$	.08		
15			$.52^{***}$	.07		
19			$.46^{***}$	.09		
21			$.74^{***}$	.05		
23			$.47^{***}$	.08		
28			$.66^{***}$	.07		
31			$.65^{***}$	.06		
			Mental Fatigue			
3	$.58^{***}$	.07			.19	.12
10			$.55^{***}$	.07		
17			$.74^{***}$	.06		
27			$.86^{***}$	.04		
32			$.72^{***}$	.05		
34			$.77^{***}$	.05		
37			$.75^{***}$	.05		

Note. Parameter estimates were derived from the final model in tests of measurement invariance (see Table 3).

$^{*}p < .05$. $^{**}p < .01$. $^{***}p < .001$.

Post hoc measurement invariance analyzes

Eleven items included categories with only one or two observations (of which all cases were among inpatients). Analysis was rerun with these categories collapsed to examine effects of low frequency cells on the estimation of parameters. Collapsing categories resulted in slight changes in fit statistics without substantial interpretational consequences.
